# Autism Spectrum Disorder in Pediatric Idiopathic Intracranial Hypertension

**DOI:** 10.3390/life11090972

**Published:** 2021-09-15

**Authors:** Anne K. Jensen, Claire A. Sheldon, Grace L. Paley, Christina L. Szperka, Geraldine W. Liu, Grant T. Liu, Shana E. McCormack

**Affiliations:** 1 Division of Ophthalmology, Children’s Hospital of Philadelphia, Philadelphia, PA 19104, USA; jensena@chop.edu (A.K.J.); claire.a.sheldon@gmail.com (C.A.S.); paleyg@wustl.edu (G.L.P.); 2 Perelman School of Medicine at the University of Pennsylvania, Philadelphia, PA 19104, USA; szperka@chop.edu (C.L.S.); mccormacks1@chop.edu (S.E.M.); 3 Department of Ophthalmology, University of British Columbia, Vancouver, BC V6T 1Z4, Canada; 4 Department of Ophthalmology, Washington University, St. Louis, MO 63110, USA; 5 Division of Neurology, Children’s Hospital of Philadelphia, Philadelphia, PA 19104, USA; liug@chop.edu; 6 Division of Endocrinology and Diabetes, Children’s Hospital of Philadelphia, Philadelphia, PA 19104, USA

**Keywords:** IIH, idiopathic intracranial hypertension, pseudotumor cerebri syndrome, autism

## Abstract

In recent years, the substantial burden of medical comorbidities in autism spectrum disorder (ASD) populations has been described. We report a retrospective observational case series of pediatric patients with suspected idiopathic intracranial hypertension (IIH) and concurrent ASD. Pediatric subjects with suspected IIH aged 2–18 years were identified by review of a pediatric neuro-ophthalmologist’s database spanning from July 1993 to April 2013. ASD diagnoses were identified within this cohort by an ICD-9 diagnosis code search and database review. Three subjects had concurrent ASD diagnoses; all were non-obese males. Since the retrospective observational case series was performed in April 2013, we identified three additional IIH cases in boys with ASD. Our experience suggests that IIH may be a comorbidity of ASD, particularly in non-obese boys.

## 1. Introduction

Autism spectrum disorder (ASD) is a neurodevelopmental condition characterized by deficits in social communication and interaction [1]. In recent years, the substantial comorbidity burden among the ASD population has become apparent, with over 70% of patients having concurrent medical, developmental, or psychiatric conditions [1].

Pseudotumor cerebri syndrome (PTCS) occurs in the setting of increased intracranial pressure with normal brain parenchyma [2]. The term idiopathic intracranial hypertension (IIH) is used when there is no evidence of a likely precipitant. Compared with adult IIH, pediatric IIH is less strongly associated with female sex predilection and obesity, and it is more likely to present asymptomatically [3,4,5,6]. Pubertal or post-pubertal IIH is more similar to adults with female sex predilection and obesity. In the course of caring for children with IIH, we noted several cases with concurrent ASD diagnoses, especially among non-obese boys.

## 2. Materials and Methods

We performed a retrospective observational chart review at our institution in 2013 as part of a multicenter study [7]. This study was approved by the Institutional Review Board at Children’s Hospital of Philadelphia (IRB# 13-010158); a subsequent case series was considered exempt from IRB review. Possible IIH subjects aged 2–18 years were identified from a pediatric neuro-ophthalmologist’s database spanning from July 1993 to April 2013. Diagnostic criteria^2^ were used to identify “definite” and “probable” cases. ASD diagnoses were identified by an ICD-9 search (299.00, 299.01, 299.80, 299.81, 299.90, and 299.91) and database and EMR review. The demographics, height and weight at the time of IIH diagnosis, and ASD diagnostic criteria and comorbidities where relevant were extracted. Pediatric obesity was defined per the US Centers for Disease Control (CDC) as BMI ≥ 95th percentile (Z-score ≥ 1.64) for the age and sex. Z-scores were assigned using the CDC 2000 growth references [8]. Once the initial retrospective record review was completed, we were then vigilant to identify more cases from 2013 to 2016.

## 3. Results

In the retrospective review, 68 cases of IIH were identified, and of these, there were 58 definite and 10 probable IIH cases. Approximately one third of the cohort was male (24/68), and about two thirds was female (44/68). Of the 24 boys, 7 (31.6%) were obese, 15 (52.6%) were non-obese, and 2 (15.8%) had missing anthropometrics. Of the 44 girls, 22 (50.5%) were obese, 16 (31.2%) were non-obese, and 6 (18.3%) had missing anthropometrics.

Three subjects with IIH had concurrent ASD diagnoses. One satisfied a definite diagnosis of IIH [2]. One other subject satisfied the criteria for a definite diagnosis, and the other was a probable diagnosis, except MR venograms were not performed, although their brain MRIs did not have any evidence of major cerebral venous occlusion. All three subjects with ASD were non-obese males (Table 1 and Table 2), and they are described below. No subjects were taking medications that would predispose them to elevated intracranial pressure.

The rate of ASD among all subjects with definite or probable IIH was 4.4% (3/68). The rate of ASD among non-obese males was between 17.6% (3/17, conservatively assuming all boys missing anthropometrics were non-obese) and 20.0% (3/15, assuming all boys missing anthropometrics were obese).

### 3.1. Case 1

Case 1 was diagnosed with ASD by the age of 2 years after a regression of language and developmental skills. He engaged in self-stimulatory behaviors, played with toys mechanically, and avoided goal-oriented or imaginary play. He displayed sensory input seeking (e.g., by squeezing into small spaces), decreased peer interactions, and obsessive compulsive and aggressive behaviors such as biting, axial hypotonia, dystonic hand posturing, difficulty sleeping, and strict adherence to routine. He developed a specific obsessive interest. In addition, he was diagnosed with epilepsy and attention deficit hyperactivity disorder (ADHD).

His neurologist proposed that his presentation included autistic features but could also be consistent with a static encephalopathy. Genetics studies did not disclose a unifying etiology. These included *CDKL5* and *POLG1* sequencing, *SCN1A* and *MECP2* sequencing and deletion or duplication testing, mitochondrial DNA genome sequencing, and transferrin isoelectric focusing for congenital disorders of glycosylation. His X-linked intellectual disability panel was notable for four potential disease-causing variants (in each of *ATRX*, *FANCB*, *ZNF81*, and *ARX*), but all were of undetermined significance. Testing of his developmentally normal mother confirmed her status as an unaffected carrier of all four alleles. His developmentally normal brother was also a carrier of the *ZNF81* allele.

At 6 years of age, he developed an intermittent esodeviation, prompting pediatric neuro-ophthalmologic consultation. During this examination, he was noted to have bilateral cranial nerve VI palsies and optic disc elevation. The MRI and MRV tests were normal, and LP under sedation revealed an elevated OP of 305 mm of cerebrospinal fluid (CSF) with a normal composition. He was underweight (BMI Z-score: −1.64). Definite IIH was diagnosed, and he was treated with oral acetazolamide and topiramate. Over time, his papilledema and esotropia improved. Repeat MRI and MRV tests were normal, and a repeat LP demonstrated a normal OP. He underwent two strabismus surgeries for residual esotropia, and at his last examination, his papilledema had resolved. The acetazolamide and topiramate were tapered.

### 3.2. Case 2

Case 2 developed some early language skills which later regressed. He was intellectually disabled and displayed pacing, mouth twitching, arching postures, and facial grimacing. Behavioral issues arose, including incontinence, spitting, and self-injury. In addition, he was diagnosed with ADHD. No information regarding genetic evaluation was noted.

At the age of 9 years, he was evaluated by our pediatric neuro-ophthalmology service for optic nerves that were abnormal in appearance, noted incidentally by his pediatric ophthalmologist. The patient had a known history of amblyopia and esotropia and had previously undergone strabismus surgery. On examination, he was found to have elevated optic nerves bilaterally. The MRI was unremarkable, and LP under sedation demonstrated an OP of 270 mm of CSF with normal constituents. Acetazolamide was initiated for IIH. His BMI Z-score at the time of diagnosis was −0.59, placing him in the normal weight range. His optic nerve swelling resolved over the ensuing months, and given his excellent response to treatment, MRV was not pursued.

### 3.3. Case 3

Case 3′s parents noticed autism-like signs around 2–3 years of age. He became nonverbal after a period of normal language development in his first year and was delayed intellectually. He displayed self-stimulatory behaviors that impacted his participation in age-appropriate relationships. He had difficulty sleeping, displayed outbursts of biting, and engaged in self-injury.

Genetic evaluation revealed a chromosome 7p22.2 deletion which he shared with his mother, who was developmentally typical. Thus, the clinical significance of this deletion was deemed uncertain. No further information regarding genetic evaluation was documented.

At the age of 5 years, he was referred to neuro-ophthalmology for evaluation of swollen optic nerves, which were noted incidentally. The examination was remarkable for bilateral optic disc elevation. The brain MRI was normal, and LP under sedation revealed an elevated OP of 310 mm of CSF with normal constituents. He was diagnosed with IIH and started on acetazolamide. He was not obese (BMI Z-score: −0.50). Over several months, his optic nerve elevation resolved, and MRV was therefore not pursued.

### 3.4. Additional Cases

Since the conclusion of our original chart review study in April 2013, we identified three additional IIH cases in boys with ASD: one obese child with probable IIH (Case 4) and two non-obese children with definite IIH (Cases 5 and 6) (Table 1 and Table 2). No subjects were taking medications that would predispose them to elevated intracranial pressure.

### 3.5. Discussion

We found that the rate of ASD among non-obese males in our retrospective pediatric IIH cohort was somewhere between 17.6% and 20.0% (depending on the obesity status of a subset of our male subjects with missing anthropometric data). These rates seem higher than would be expected from ASD general population estimates (overall: 0.62–2%), even when accounting for the asymmetry of disease burden in males (3.24–3.74%) over females (1.30–1.47%) [9,10].

Our findings suggest a possible association between ASD and pediatric IIH, particularly among non-obese males. Children with ASD could possibly have as of yet uncharacterized underlying bioenergetic, genetic, metabolic, or hormonal susceptibilities to IIH, and future prospective mechanistic studies should address this possibility, since it could have implications for diagnosis and management.

The major limitation of our study is the case series format. Establishing a true association would require a case–control study. Furthermore, we did not test all of our patients with IIH for ASD, our patients did not systematically undergo rigorous neuropsychological testing, and we did not ascertain the frequency of IIH in ASD patients seen in our institution.

As with many ASD comorbidities, IIH may be under-diagnosed in the pediatric ASD population, especially since these patients may have difficulty reporting symptoms and cooperating with an ophthalmologic examination. Clinicians caring for children with ASD should be mindful of the possibility of an IIH comorbidity, especially if unexplained vision loss, esotropia, or protracted headaches occur.

## 4. Conclusions

We report a retrospective observational case series of pediatric patients with suspected idiopathic intracranial hypertension (IIH) and concurrent ASD. Our experience suggests that IIH may be a comorbidity of ASD, particularly in non-obese boys.

## Figures and Tables

**Table 1:** IIH diagnostic criteria for six cases of concurrent ASD and IIH.

	Case 1	Case 2	Case 3	Case 4	Case 5	Case 6
IIH Diagnosis	Definite	Probable (MRV Not Pursued)	Definite (MRV Not Pursued)	Probable	Definite	Definite
IIH Diagnostic Criteria						
**1. Papilledema**	Yes	Yes	Yes	Yes	Yes	Yes
**2. Normal Neurologic Exam** (except for cranial nerve abnormalities)	Yes	Yes	Yes	Yes	Yes	Yes
**3. Normal MRI**	Yes	Yes	Yes	Yes	Yes	Yes
**4. Normal MRV** (required for atypical (i.e., not female and obese) patients)	Yes	Not Pursued	Not Pursued	Yes	Yes	Yes
**5. Normal CSF Composition**	Yes	Yes	Yes	Yes	Yes	Yes
**6. Elevated OP** (>/= 280 or >/= 250 mm CSF if non-obese and not sedated)	Yes	No (OP = 270 mm CSF under sedation)	Yes	No (OP = 275 mm CSF under sedation)	Yes	Yes

Definite diagnosis = all criteria met. Probable diagnosis = all criteria met except for elevated OP on LP. Autism spectrum disorder (ASD), cerebrospinal fluid (CSF), idiopathic intracranial hypertension (IIH), lumbar puncture (LP), magnetic resonance imaging (MRI), magnetic resonance venography (MRV), and opening pressure (OP).

**Table 2:** Characteristics of six cases of concurrent ASD and IIH.

Characteristics	Case 1	Case 2	Case 3	Case 4	Case 5	Case 6
**Gender**	Male	Male	Male	Male	Male	Male
**Age at IIH Diagnosis (Years)**	6.1	10.7	5.6	12.2	7.9	7.9
**BMI at IIH Diagnosis (kg/m^2^)**	13.73	15.30	14.81	29.11	17.12	18.37
**BMI Z-score at IIH Diagnosis**	−1.64	−0.59	−0.50	2.16	0.73	1.21
**Obesity Status**	No	No	No	Yes	No	No
**Co-morbidities**	• Intellectual disability • Language disorder • Sleep disturbance • Aggressive behavior • ADHD • OCD • Epilepsy • Otitis media	• Intellectual disability • Language disorder • Sleep disturbance • Aggressive behavior • Self-injurious behavior • ADHD • Gastrointestinal problems • Cryptorchidism	• Intellectual disability • Language disorder • Sleep disturbance • Aggressive behavior • Self-injurious behavior • Gastrointestinal problems • Abnormal eating • Horseshoe kidney	• ADHD • Vitamin D deficiency	• Premature pubarche • OCD • Recurrent perianal streptococcal infection	• Language disorder • OCD • Hearing loss

## Data Availability

We do not have any publicly archived datasets.

## Meeting Presentation

Presented at the North American Neuro-Ophthalmology Society (NANOS) 2015 meeting in San Diego; California and presented at the Scheie Alumni 2015 meeting in Philadelphia, Pennsylvania.
